# Evolution of drug resistance in HIV protease

**DOI:** 10.1186/s12859-020-03825-7

**Published:** 2020-12-30

**Authors:** Dhara Shah, Christopher Freas, Irene T. Weber, Robert W. Harrison

**Affiliations:** 1 Department of Computer Science, 25 Park Place, Atlanta, GA 30303 USA; 2 Department of Biology, 100 Piedmont Ave., Atlanta, GA 30303 USA

**Keywords:** HIV protease, Drug resistance, Machine learning, Evolution, Structure-based

## Abstract

**Background:**

Drug resistance is a critical problem limiting effective antiviral therapy for HIV/AIDS. Computational techniques for predicting drug resistance profiles from genomic data can accelerate the appropriate choice of therapy. These techniques can also be used to identify protease mutants for experimental studies of resistance and thereby assist in the development of next-generation therapies. Few studies, however, have assessed the evolution of resistance from genotype–phenotype data.

**Results:**

The machine learning produced highly accurate and robust classification of resistance to HIV protease inhibitors. Genotype data were mapped to the enzyme structure and encoded using Delaunay triangulation. Estimates of evolutionary relationships, based on this encoding, and using Minimum Spanning Trees, showed clusters of mutations that closely resemble the wild type. These clusters appear to evolve uniquely to more resistant phenotypes.

**Conclusions:**

Using the triangulation metric and spanning trees results in paths that are consistent with evolutionary theory. The majority of the paths show bifurcation, namely they switch once from non-resistant to resistant or from resistant to non-resistant. Paths that lose resistance almost uniformly have far lower levels of resistance than those which either gain resistance or are stable. This strongly suggests that selection for stability in the face of a rapid rate of mutation is as important as selection for resistance in retroviral systems.

## Background

Selection pressure due to the widespread use of anti-retroviral therapy [1] makes Human Immunodeficiency Virus (HIV) a valuable model for studying evolution. HIV/AIDS is a major pandemic disease [2] where more than 37 million people have been infected. Currently, about 60% of the infected people receive anti-retroviral therapy. Antiviral drugs block viral replication by targeting the viral enzymes, protease, reverse transcriptase and integrase, HIV entry and fusion to the host cell. Further progress in treating the disease is hampered by the selection of drug-resistant viral strains. Since the conversion from the RNA genome to DNA is error-prone, HIV mutates rapidly [3]. HIV readily forms quasi-species and distinct viral strains. Thus, HIV possesses the prerequisite high degree of variation for the rapid evolution of drug resistance. In addition to studying drug resistance to enhance the development of novel therapeutics, studying the evolution of drug resistance can help define the optimal strategy to overcome drug resistance with current approaches.

HIV protease is an excellent model system due to its relatively small size and the extensive data for sequence variants and structures [4]. The protease acts as a dimer of two 99-residue subunits. Experimental studies [5] and theoretical analysis [6] of the protease mutants suggest that many of the secondary mutations contribute to the survival of the original resistance mutations by improving the effectiveness of the protease for viral replication. These findings suggest that initial mutations introduce resistance and further selection improves the fitness of the enzyme. Therefore, we expect to see linked sets of mutations in the resistance data, and have developed an analysis based on Minimum Spanning Trees to detect and analyze these linkages.

Previous studies by our group and others show that machine learning can accurately predict resistance phenotype from genotype data for HIV protease and reverse transcriptase [7–14]. We have found that including structural data with the sequence using Delaunay triangulation is an especially effective representation for machine learning [15]. The combined sequence and structure information is compressed into a single 210-dimension vector for each mutant. In essence, this approach is an sequence edit distance weighted by the most significant local contacts in the protein. It is nearly a linear metric space [11]. Simple machine learning approaches such as a linear SVM and k-nearest neighbors are able to reliably classify resistance data with this encoding of sequence and structure. This approach is a marked contrast from other work where complicated or deep machine learning approaches are used [13, 14, 16]. It creates the ability to use the features for more than simple classification or regression.

Our previous work [7–9, 11, 15, 17] has concentrated on developing models for predicting the resistance to single inhibitors. Shen et al. [11] and Pawar et al. [17] demonstrated classification accuracies higher than 99%. However, many of the resistant strains have lost susceptibility to all clinical inhibitors. It is important, therefore, to apply machine learning to the prediction of resistance to multiple inhibitors. Our previous work [17] showed that there was significant cross-prediction accuracy where models trained on one inhibitor predict the response to other inhibitors. This suggests that there are commonalities in resistance mechanisms and the first step to studying these commonalities is to build a machine learning model that describes them. This model can then be used to select sequences for expression, characterization, and structural analysis.

Gene trees are a major tool in the construction of molecular phylogeny [18–21] and they have been applied to HIV [22]. Much of the existing work has been applied to estimate gene flow between species, gene duplication, and horizontal transfer. Typically, sequence distances are used to estimate similarity between genes and then a graph is constructed that reflects the relationships between the genes. The graph is a tree in the absence of horizontal transfer and gene duplication. There are subtle but important differences between the standard use of gene trees and this study because mutational data in the HIV protease gene do not involve gene flow between species, gene duplication or horizontal transfer. This paper examines the onset of speciation or quasi-speciation under the selection pressure of clinical treatment with potent protease inhibitors. It combines our highly effective representation of structure and sequence with well-understood algorithms for building minimum spanning trees (MSTs) to estimate the evolutionary properties of HIV response to drugs. Since this measure is linear or nearly linear and possesses metric properties, it should be an effective proxy for evolutionary distance. MSTs will serve as a first approximation to the gene tree.

The development of “super-resistance” is a related question. Naive selection theory suggests the “first past the post” mutations, those that are sufficiently resistant to allow HIV to reproduce in the presence of inhibitors, will be a majority of those selected. If drug resistance alone is sufficient for evolutionary selection, why should new mutations accumulate in the protease? Yet there are many examples of highly resistant proteases bearing different sets of multiple mutations which are believed to enhance viral replication [23]. The pattern of resistance acquisition and loss along branches of the MSTs sheds light on the selective pressures for drug resistance. The virus must not just become resistant, but must retain resistance and effective replication in the presence of a high mutation rate.

### Genotype–phenotype data from the Stanford HIVdb

The collated data in the Stanford database [24] is a valuable resource for computational analyses. The data consist of the sequences of HIV drug targets, including HIV protease, and resistance measures. The database is curated and updated regularly to reflect the current status of drug resistance in HIV. We used the filtered phenosense data for this paper [25].

## Results

### Resistance classification and regression

The linear SVM was used to classify if the HIV protease sequence is resistant or not based on the threshold of 3.0 as defined in the Stanford database (shown in Table 1). The data are well-balanced for all inhibitors with the exception of Darunavir. Both the SWED and RSWED were used to train two different models with threefold cross validation. The quality of the prediction shows that our data were successfully updated. Table 2 shows the classification accuracy for pairs of inhibitors. Note that while there is some correlation between different inhibitors, there are significant differences between them. Table 3 shows the results for triples of inhibitors. Only a subset (those with ATV) is shown to conserve space, but the results are similar for all triples with both the RSWED and SWED metrics.

**Fig. 1:**
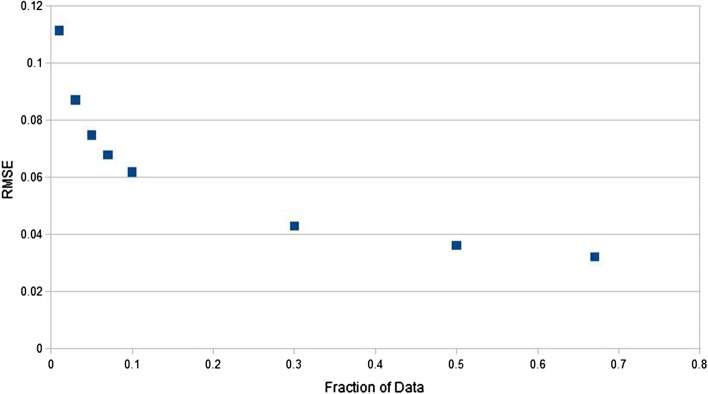
The dependence of RMSE on the fraction of data used to train a regression analysis for one inhibitor based on the SWED encoding

**Fig. 2:**
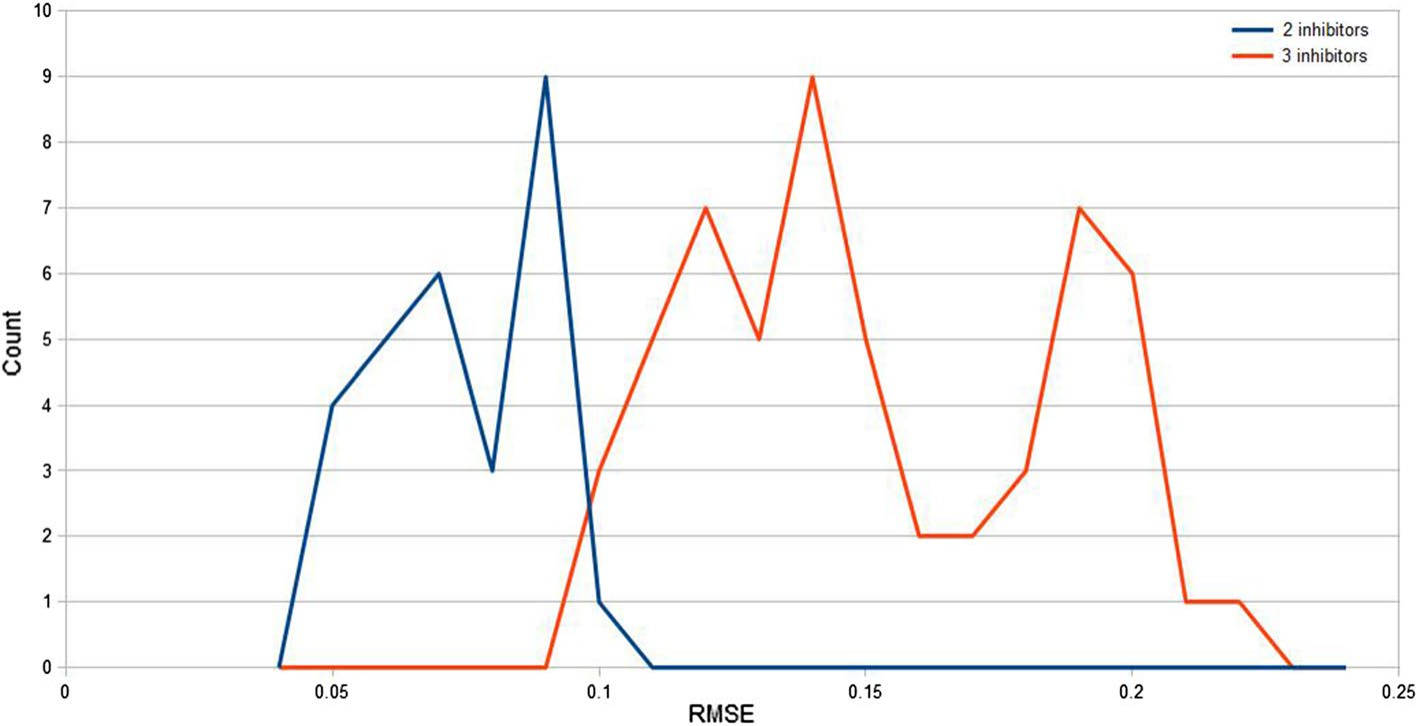
The distribution of RMSE between calculated and observed resistance values in 2 and 3 inhibitor regression analyses. For two inhibitors the RMSE ranges from 0.04 to 0.1 and for three inhibitors from 0.1 to 0.22

In addition to single inhibitors, classification of the resistance for all pairs and triples of inhibitors was done. In all cases, a high classification accuracy (${>}\,98\%$) was seen. Therefore, it was important to examine regression, where the magnitude of the observed effect is predicted. This is a more difficult measure than binary classification. Regression was preformed using random forest regression. Figure 1 shows the RMSE as a function of the training fraction for cross-validation. A training fraction of 0.66 corresponds to threefold cross validation (2:1 ratio) and 0.2 is an inverted fivefold cross validation (1:4). Since the range of observed values for the data is between 0 and 100, an RMSE ${<}$ 0.1 corresponds to a high degree of accuracy. Figure 2 shows the distribution of RMSE for regressions over each pair and triple of inhibitors. The correlation coefficients were in the high range from 98 to 99%.

#### Spanning trees

**Fig. 3:**
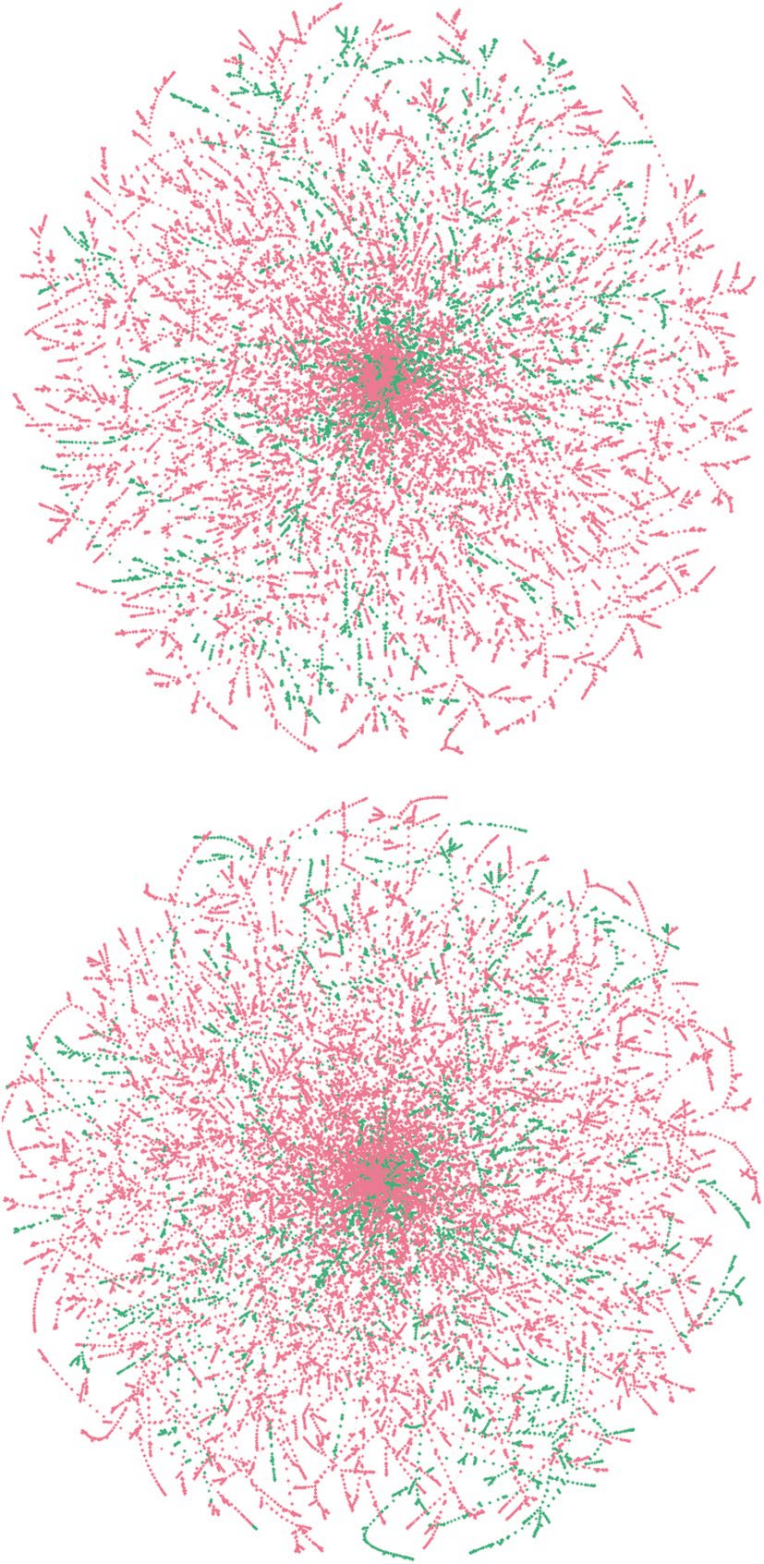
*l*2 norm spanning trees of ATV resistance. The upper panel shows the distance based sums (SWED) and the lower panel shows the count based sums (RWSED). Resistant and non-resistant nodes are represented by green and red colors respectively

Figure 3 represents the spanning trees of a random 10% split of data with ATV. The nodes in these graphs represent the vectors generated by the upper triangular matrix with average distance and count, respectively. These spanning trees are calculated with respect to $l_2$ distances when the nodes are represented by distance and count vectors, respectively. The nodes that are resistant with value bigger than 3 for inhibitor are represented as green, and the non-resistant nodes are represented as red. Empirically, the spanning trees for all splits with respect to all the inhibitors have similar visualizations. The centers of these trees are the nodes whose sequences differ at most in two places from the standard wild type HIV-1 protease sequence of the group B sub-type M. Consistent with the high mutational rate of HIV, both resistant and susceptible strains develop differences from the standard sequence in a similar manner.

#### Path statistics in the spanning trees

Since the paths or branches in Fig. 3 appear to show the selection for resistance early in mutational history, followed by its conservation over time, it is necessary to examine the behavior of resistance along the branches of the tree. The sequences at the roots of the tree are close to the reference sequence and the branches, both resistant and non-resistant, show increasing numbers of mutations as they move from away from the center. The paths fall into five general categories, those that: remain below the resistance threshold, gain resistance, lose resistance, remain above resistance threshold, or cross the threshold multiple times, creating a spiking pattern.

Due to the density of the data, we summarized the gain, loss and spike patterns by plotting the mean value of resistance against the fraction of the path which is above the resistance threshold. Figures 5, 6 and 7 represent the scatter plots of paths that gain, lose or spike in resistance. Each dot in these figures corresponds to an individual path. Figure 8 shows the histogram of the variance of paths above the resistance threshold. Most of the paths that are resistant have low variance,s which indicates that the magnitude of the resistance is stable, and therefore there is selection for stable resistance in the presence of high mutational rates.

## Discussion

This paper demonstrates three points. First, it shows that SWED and RSWED measures still work well for classification and regression of resistance. This result is important since the sequence-structure representation was recalculated when the database was updated. Second, it shows that these representations, when used to generate an MST, appear to be valid proxies for evolutionary or mutational distances. Finally, the trajectory of resistance along individual branches of the trees suggests that the selection pressures for resistance are more complicated than would be naively thought.

### Classification and regression

With an elegant encoding, in this case the SWED and RSWED, even simple shallow learning algorithms like the SVM can achieve high accuracy. The accuracy in this paper is better than we achieved earlier, and we hypothesize that this is due to using better and more complete data. Including features of the geometry (amino acid positions), along with the labels (the sequence), results in an encoding for a physical object that captures most of the essential information.

### A proxy for evolutionary distance

Defining the evolutionary distance between two individual genomes is an open problem. Obviously, the distance must reflect mutations, but in a highly mutable system like HIV, straightforward counts of mutations can be misleading because the probability of a reverting mutation is relatively high. Therefore, including structural or biochemical information to assess the importance of individual mutations should improve accuracy. The SWED and RSWED measures include structural information. Figure 3 shows a visualization of the MST derived with both measures. The sequences at the roots of the tree are close to the reference sequence, while the branches, both resistant and non-resistant, show increasing numbers of mutations as they move away from the center. Interestingly, many of the branches maintain resistance or non-resistance during evolution. Quite often an initial single or double mutation becomes resistant and the resistance evolves further with additional mutations.

### Behavior of the branches

Analysis of the branches shows several interesting results. Most importantly, it shows that selective pressure for resistance is complicated. Figures 5, 6 and 7 show the relationship between path length and resistance for both paths that gain resistance and those that lose it. The naive model of selection would expect that viruses would evolve to be just resistant enough to replicate in the presence of inhibitors. Resistance along a branch or path shows significant differences from this naive model. Paths that maintain resistance tend to increase resistance to high levels. However, some paths may demonstrate “spiking” where they become highly resistant and then approach lower resistance levels. Paths that lose resistance inevitably are never highly resistant. This result strongly suggests that there is an additional selective pressure to become highly resistant. In the presence of high mutation rates, molecules that are “just resistant enough” will readily lose resistance. Proteases that are highly resistant could require many mutations to lose resistance.

**Fig. 4:**
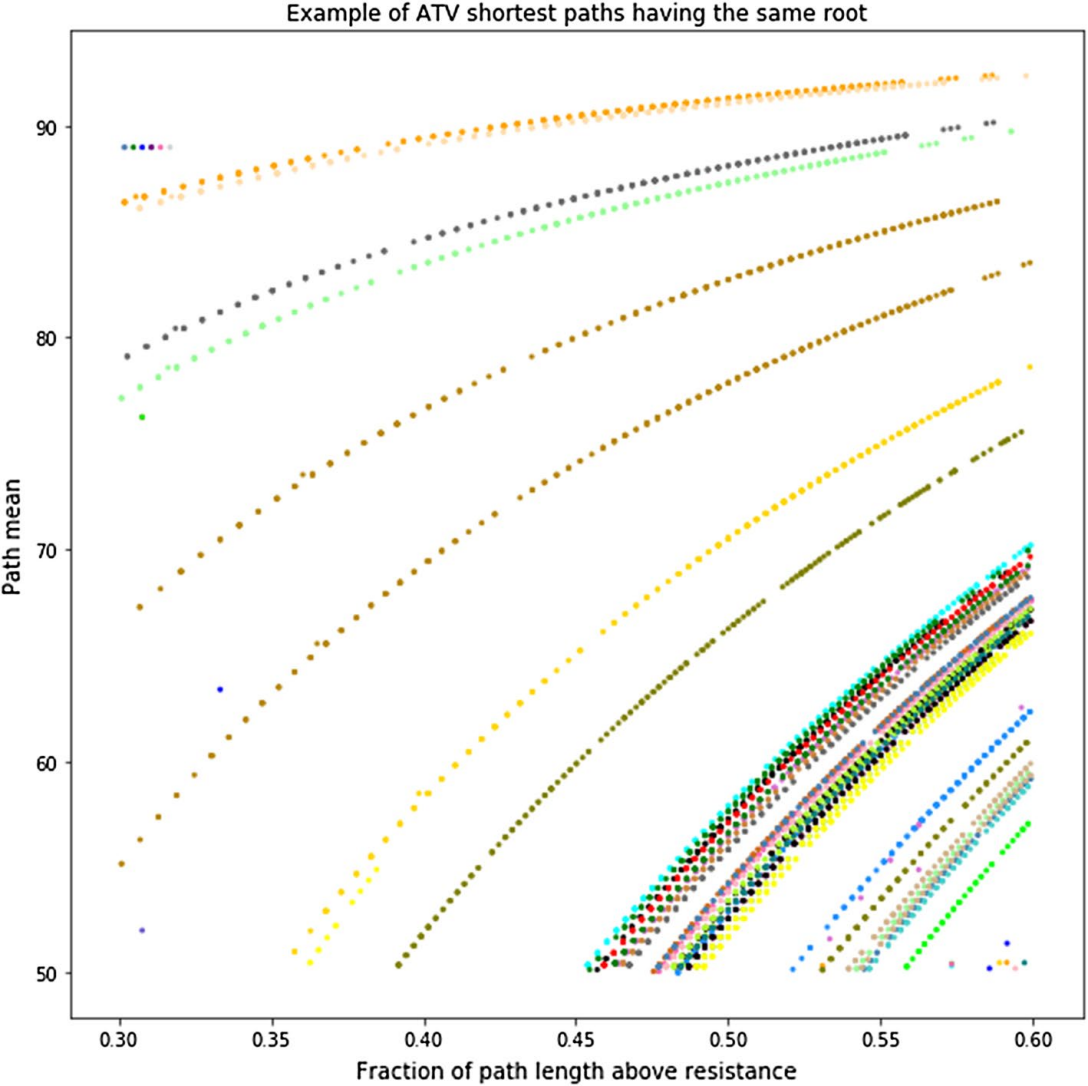
Grouping paths with the same root together in a sample of SEWD shortest paths for ATV. The y axis shows the number of paths in each cluster and the x axis shows the fraction of those paths that are above the resistance threshold

**Fig. 5:**
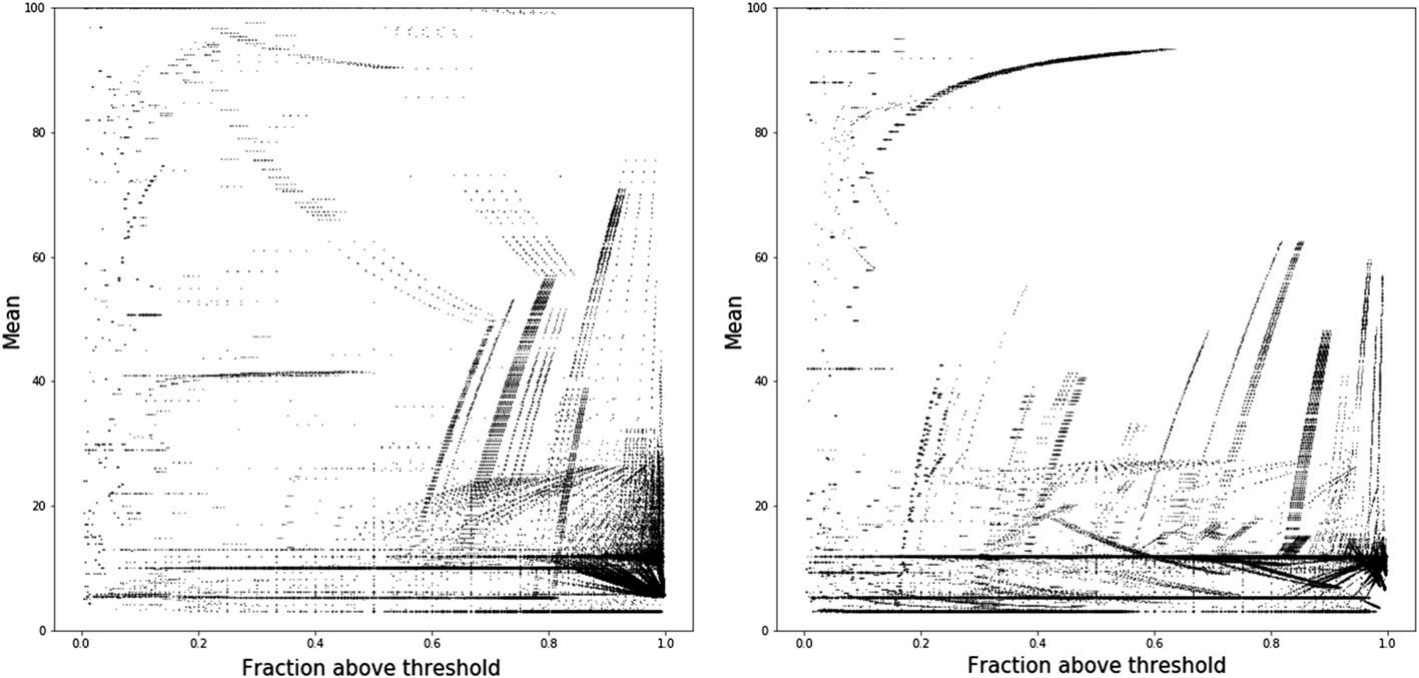
SWED (left) and RSWED (right) shortest paths that gain resistance for ATV. The y axis shows the mean value for resistance along the path and the x axis shows the fraction of the path above the threshold for resistance

**Fig. 6:**
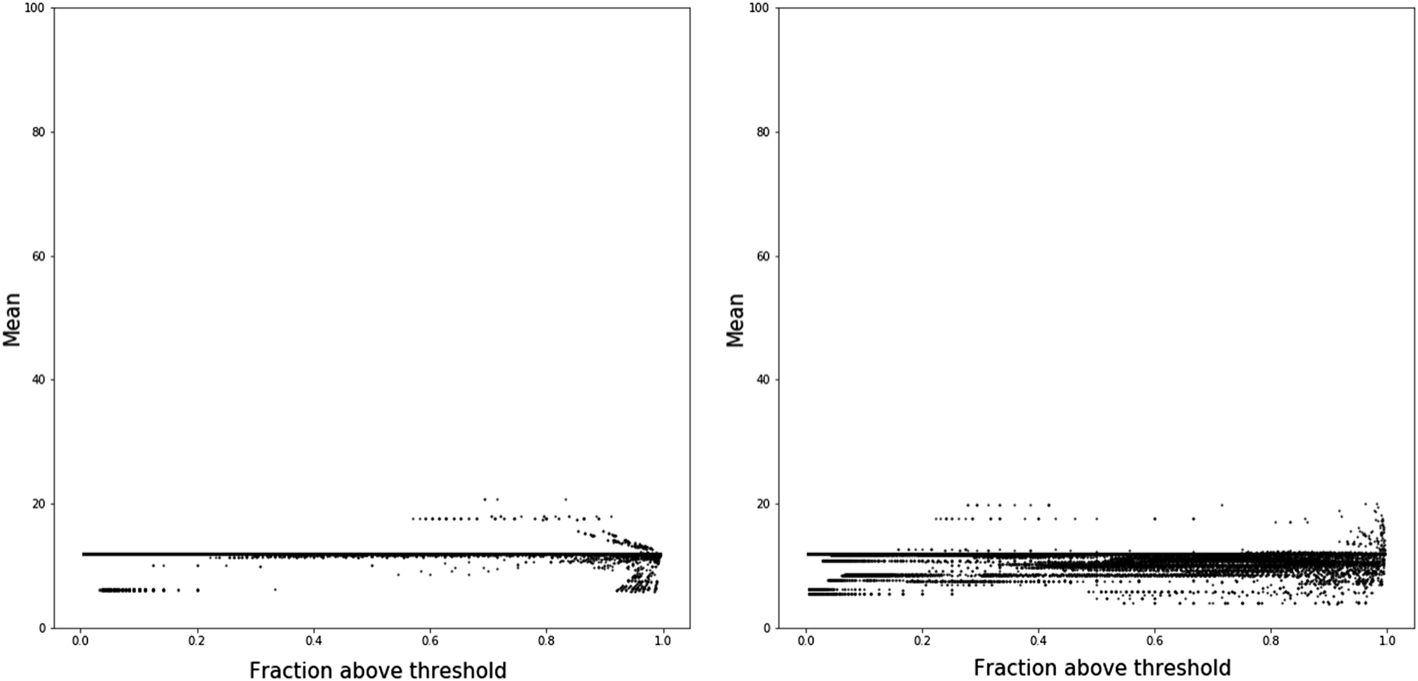
SWED (left) and RSWED (right) shortest paths that lose resistance for ATV. The y axis shows the mean value for resistance along the path and the x axis shows the fraction of the path above the threshold for resistance

**Fig. 7:**
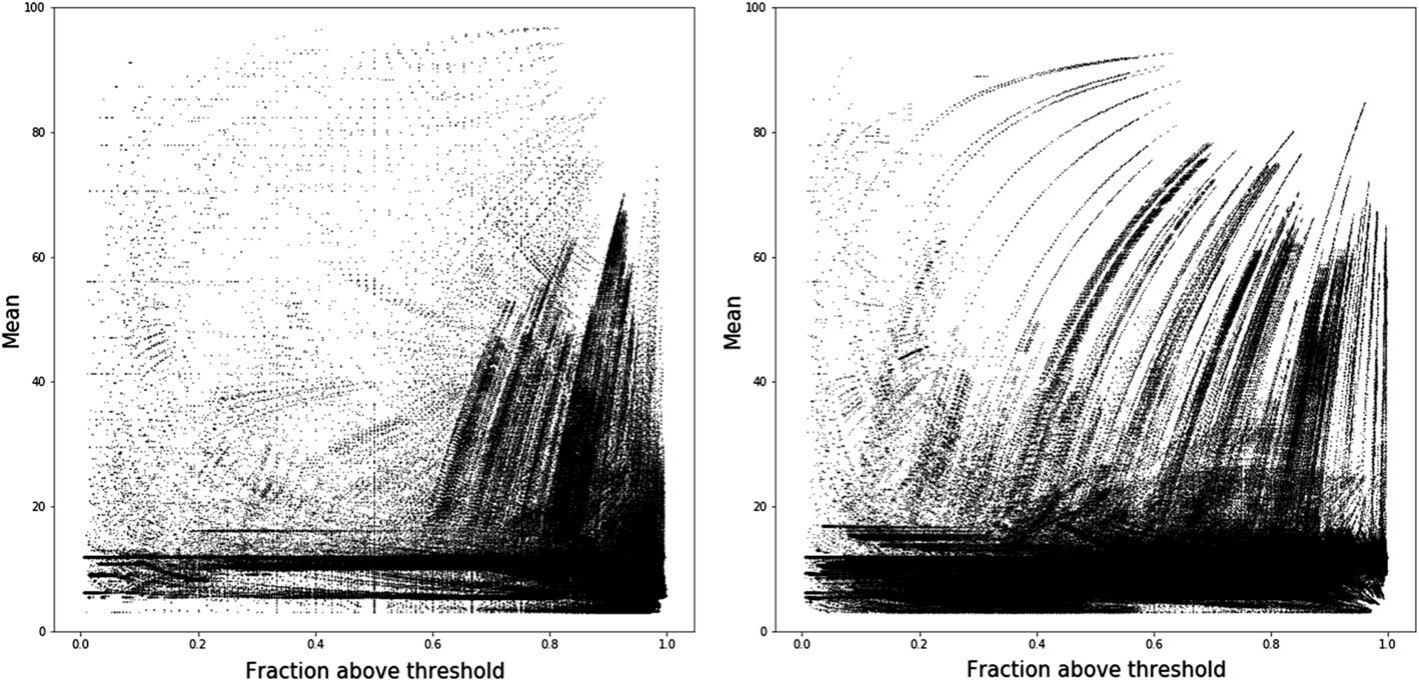
SWED (left) and RSWED (right) shortest paths that fluctuate in resistance for ATV. The y axis shows the mean value for resistance along the path and the x axis shows the fraction of the path above the threshold for resistance

It is clear in Figs. 5, 6 and 7 that there is some structure to the relationship between resistance and path length. The structure could reflect paths that have the same root and diverge at some time during viral evolution. As a first pass at analyzing this relation ship, we clustered paths using the dbscan [26] algorithm as implemented in python scikit learn [27] library. The similarity of paths starting from the same root is shown for a representative sample in Fig. 4. That these points appear to lie on smooth curves suggests that the structure seen in Figs. 5, 6 and 7 is due to paths that diverge during evolution.

## Conclusion

A simple measure that combines structure and sequence is highly effective for classification and regression of drug resistance in HIV protease. Unlike pure sequence features, shallow learning, even simple shallow learning algorithms like the linear SVM, produce accurate results with this representation. In addition to clustering and selecting sequences for experimental study, the measure can be used for calculations the probe the evolutionary relationship between isolates of HIV. Our results suggest two major points for evolution of resistance. First, there is a conservation of resistance. Isolates become resistant early on and then tend to stay resistant. Second, there is a selective pressure for isolates to become highly resistant over time. Isolates that do not become highly resistant tend to lose resistance. This suggests that robustness with respect to mutation and change is an important selection pressure in evolution.

**Table 1:** Classification statistics for HIVpr

Inhibitor	Fraction resistant	Fraction susceptible	Accuracy	F-score
FPV	36.4	63.6	99.5	99.5
ATV	21.5	78.5	99.7	99.8
IDV	33.8	66.2	99.6	99.7
LPV	34.5	65.5	99.6	99.6
NFV	27.3	72.7	99.6	99.7
SQV	68.3	31.7	99.6	99.6
TPV	60.4	39.6	99.7	99.8
DRV	97.1	2.9	99.92	99.93

Fraction of resistant vs non-resistant inhibitors for all inhibitors. Other than for Darunavir, the datasets are well-balanced. The threefold cross-validated accuracy and F-scores are shown for the count vectors using a linear SVM

**Table 2:** Classification statistics for pairs of inhibitors HIVpr using the SWED metric

Inhibitor	Inhibitor	Pearson R	Accuracy
ATV	DRV	0.6444	99.8577
ATV	IDV	0.6139	99.7566
ATV	LPV	0.3535	99.8217
ATV	NFV	0.8655	99.808
ATV	SQV	0.9175	99.796
ATV	TPV	0.3837	99.808
FPV	ATV	0.617	99.8168
FPV	DRV	0.8092	99.8715
FPV	IDV	0.8215	99.8682
FPV	LPV	0.8694	99.8907
FPV	NFV	0.7385	99.8156
FPV	SQV	0.4485	99.8219
FPV	TPV	0.4229	99.894
IDV	DRV	0.4766	99.6974
IDV	LPV	0.8671	99.7512
IDV	NFV	0.8189	99.6915
IDV	SQV	0.4657	99.6959
IDV	TPV	0.4373	99.8133
LPV	DRV	0.3024	99.8013
LPV	NFV	0.6433	99.7536
LPV	SQV	0.1664	99.7166
LPV	TPV	0.3573	99.8534
NFV	DRV	0.527	99.7646

The Pearson R is between the resistance of the two inhibitors. The threefold cross-validated accuracy is shown for random forest

**Table 3:** Classification statistics for a subset of the triples of inhibitors HIVpr using the SWED metric

Inhibitor	Inhibitor	Inhibitor	Accuracy
ATV	DRV	FPV	99.8152
ATV	DRV	IDV	99.8365
ATV	DRV	LPV	99.8519
ATV	DRV	NFV	99.8358
ATV	DRV	SQV	99.8283
ATV	DRV	TPV	99.8183
ATV	FPV	IDV	99.7601
ATV	FPV	LPV	99.7686
ATV	FPV	NFV	99.7516
ATV	FPV	SQV	99.7658
ATV	FPV	TPV	99.7919
ATV	IDV	LPV	99.8149
ATV	IDV	NFV	99.8037
ATV	IDV	SQV	99.7727
ATV	IDV	TPV	99.842
ATV	LPV	NFV	99.7905
ATV	LPV	SQV	99.8075
ATV	LPV	TPV	99.8272
ATV	NFV	SQV	99.7558
ATV	NFV	TPV	99.8016
ATV	SQV	TPV	99.7983

The threefold cross-validated accuracy is shown for random forest

**Fig. 8:**
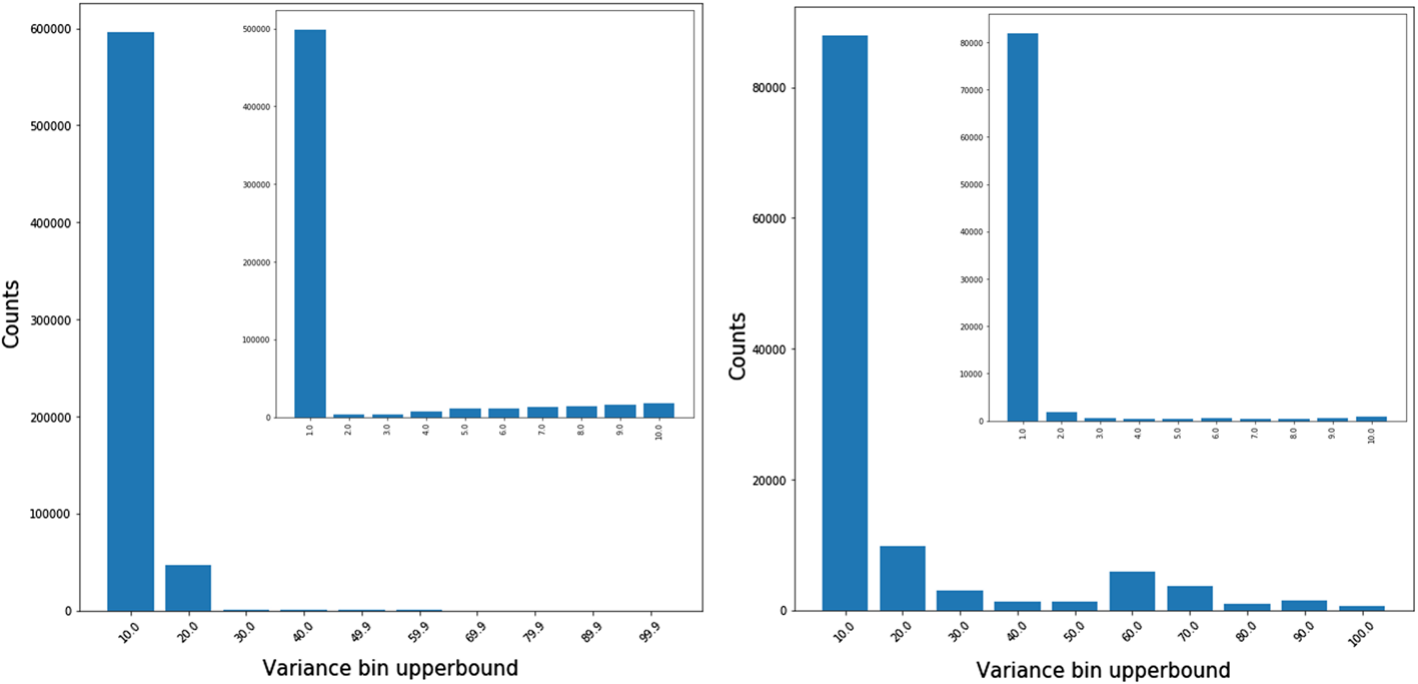
SWED (left) and RSWED (right) histograms of the shortest paths that are above resistance for ATV. 1.16% of SWED and 6.8% of RSWED paths have variance greater than 100. The histogram of paths forming the first bin are depicted in the top right corner of each figure

## Methods

The methods in this paper range from preparing the data (Data Expansion and Vector Generation) to machine learning (Classification and Regression) and the development of models of evolution. A new expansion of the data was needed as the Stanford database was updated from the version used in previous work. This was a major update where the curators cleaned up the data. Because new data were generated it was necessary to show that our methods still worked. Classification and regression showed that the machine learning approaches are still highly effective. MSTs and analysis of the branches or paths in the trees was performed to inform hypotheses about selection due to drug pressure in HIV. Our software is available from a Github repository [28]. The expanded dataset, even when compressed, was too big for the repository and will be made available upon request to qualified researchers.

### Data expansion

The Stanford dataset [24] for HIV protease is comprised of different protease sequences with the observed resistance in the Phenosense assay [25] for the 8 clinical protease inhibitors FPV, ATV, IDV, LPV, NFV, SQV, TPV and DRV. The sequence of the 99-amino acid protease monomer is presented, indicating those amino acids that are different from the consensus sequence of HIV-1 Group M subtype B. Each position in the sequence data may have more than one possible amino acid mutation. These mutations are listed as multiple abbreviations along with insertion * and deletion # for the field of that position. Sequences with two or more alternate amino acids at a single position were expanded by constructing all possible sequences as described in [7, 8]. A total of 1951 genotype sequences were expanded to 414,010 single sequences. The expansion potentially could cause “cross talk” where one member of the expansion is in the test set and another in the training set. We have shown previously that this has an insignificant effect [7, 8].

#### Vector generation

Vectors were generated for each sequence by obtaining the neighbors of each position of this sequence from the Delaunay triangulation as was done in [7–9, 11, 15, 17]. The coordinates of the $\alpha$carbon atoms were used, and all arcs of the triangulation were used. Earlier studies in our lab [7, 15] showed these were sufficient. The long arcs in the Delaunay triangulation, which correspond to distal surface contacts, are a small subset of the total set of arcs. Other coarse representations of amino acids, such as center of mass, can be highly variable with changes in the kind of amino acid. The first step in this process was to use the positions of each amino acid residue from a crystal structure of the HIV protease dimer with 198 residues (pdb entry 3oxc was used [29]). The Delaunay triangulation was generated exactly once according to the position coordinates obtained from this file and then we obtained the neighbors for each sequence based on this adjacency matrix. A $20\times 20$ amino acid matrix was generated from this adjacency matrix in two different ways: average distance and count between neighboring amino acids. Since this matrix is symmetric, we take the upper triangular values of this matrix as a vector, which is of the size $1\times 210$. The count defines a Structure-Weighted Edit Distance (SWED) and the average distance defines a Radial Structure-Weighted Edit Distance (RSWED).

#### Classification and regression

The Stanford database curators recommend a resistance value of 3 in the phenosense assay as the threshold for resistant/non-resistant proteases [24] and we used their recommendation. As a control, since we have recalculated the vectors with new data, the classification calculations were repeated. The values for threefold cross validation are shown in Table 1 and demonstrate that the data were generated successfully. The RMSE for regression for one inhibitor as a function of the size of the training set is shown in Fig. 1. This corresponds to a correlation coefficient of ${>}\,99\%$.

In addition to control calculations for single inhibitors, the same calculations were performed for all pairs and triples of inhibitors. The average classification accuracy is ${>}\,99\%$ and the distribution of RMSE is shown in Fig. 2.

Calculations were performed in python using scikit-learn [27]. Regression was done with random-forest regression using two trees. Classification used a linear SVM. Accuracy and F-Score are reported. The F-Score controls for population effects.

$$\begin{aligned} Accuracy&= \frac{TP+TN}{TP+TN+FP+FN} \\ Precision&= \frac{TP}{TP+FP} \\ Recall&= \frac{TP}{TP+FN} \\ F{\hbox {-}}Score&= 2\frac{Precision*Recall}{Precision+Recall} \\ \end{aligned}$$

where TP is true positive, TN true negative, FP false positive, and FN false negative.

#### Spanning trees for evolution prediction

Minimum spanning trees were generated for both the SWED and RSWED vectors using Python networkX [30] 2.2 and visualized with Gephi [31] 9.2. However, the amount of data forced us to use a 10% subset of the data due to limitations of the networkX library. Therefore we repeated the calculation using 10 randomly selected 10% samples from the data to ensure that the results did not depend on the particular random sample. Nodes with ‘NA’ resistance values (which were not observed or determined) were removed while making the spanning tree for each inhibitor. Spanning trees were calculated for of each of these splits. Computing spanning trees of the complete graph is computationally expensive and time consuming, hence we used the spanning tree of each split with edges connecting 400 nearest neighbors for each node. Empirically we have observed that this method yields only up to 2% different edges of resulting spanning trees, when calculated 400 nearest neighbors vs complete graphs on these splits.

#### Shortest paths from roots to leaves in the spanning trees

The roots of this spanning trees are the nodes representing sequences with low numbers of differences from the consensus “wild type” sequence of HIV-1 Group M sub-type B protease. The root nodes are same as or differ by at most two changes from the consensus sequence. We then calculate shortest paths from these nodes to all the leaves in the spanning trees. The spanning trees created by Gephi [31] 9.2 where visualized with Forced Atlas-2 [32] using a layout gravity of 35, node and edge size of 10. We have verified that the visualizations look very similar for all other inhibitors.

#### Shortest paths classification

As noted in the results, the majority of the shortest paths in these spanning trees have sequences with resistance levels that are not monotone from root to leaves. However, we are interested in the behavior of sequences that gain resistance. Hence we classify the shortest paths in four categories: paths that remain below, paths that remain above resistance level, paths that gain resistance, and paths that lose resistance. We use the direction from root to leaf as the progression for inhibitor resistance values.

#### Measurement of the resistance variance for resistant path segments

We are interested in the behavior of shortest path segments that are above resistance, namely, how does the resistance level vary when the nodes in the path are resistant. In order to understand this, we calculated the fraction of the path above resistance and the variance of the resistance values for these path nodes.

## Data Availability

The unprocessed datasets can be downloaded from: http://hivdb.stanford.edu/pages/genopheno.dataset.html.

## Abbreviations

ATV: Atazanavir
DRV: Darunavir
FPV: Fos-Amprenavir
IDV: Indinavir
LPV: Lopinavir
NFV: Nelfinavir
SQV: Saquinavir
TPV: Tipranavir
PPV: Positive predictive value
HIV: Human immunodeficiency virus
HIVpr: HIV protease
SWED: Structure weighted edit distance
RSWED: Radial structure weighted edit distance
SVM: Support vector machine
